# Supplementary material for: Cellular stress signaling activates type-I IFN response through FOXO3-regulated lamin posttranslational modification
Source: Nat Commun. 2021 Jan 28;12:640. doi: 10.1038/s41467-020-20839-0 (PMC7843645; doi:10.1038/s41467-020-20839-0)
Supplement: Supplementary file 1 — Supplementary Information [file 41467_2020_20839_MOESM1_ESM.pdf]

Cellular Stress Signaling Activates Type-I IFN Response Through FOXO3-regulated Lamin Posttranslational Modification

Inah Hwang, Hiroki Uchida, Ziwei Dai, Fei Li, Teresa Sanchez, Jason W Locasale, Lewis L Cantley, Hongwu Zheng, and Jihye Paik

Supplementary figure 1-9

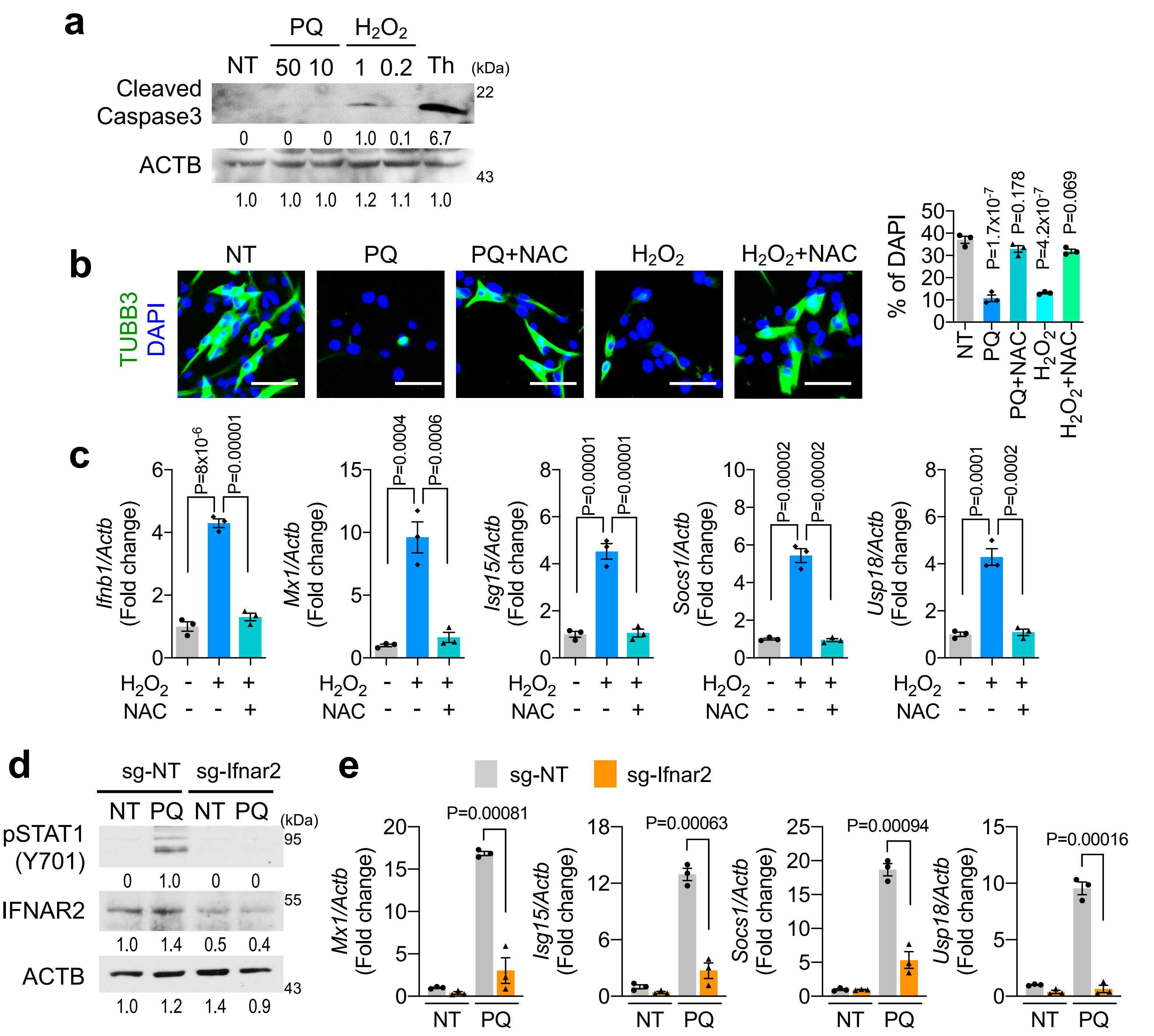

Supplementary figure 1. Redox stress suppresses neuronal differentiation through IFN-I signaling. a. Representative WB results for indicated proteins following 2 days of PQ (50, 10 μM), H_2O_2 (1, 0.2 mM), or Thapsigargin (Th, 1μM) treatment. **b**. Representative IF images of TUBB3 on 3 day of differentiation following 48h of indicated treatments. Scale bar= 50 μm. % of TUBB3-positive cells is plotted on right. Mean ± s.e.m. of three independent experiments. Each p value was determined by one-way ANOVA when compared to NT group. **c.** qRT-PCR results following 4 days of indicated treatments. Mean ± s.e.m. of three independent experiments. **d.** Representative WB results for indicated proteins following 2 days of PQ (10 μM) treatment in NSPCs expressing guide RNA non-targeted (sg-NT) or targeting Ifnar2 (sg-Ifnar2). **e**. qRT-PCR results following 4 days of PQ treatment. Mean ± s.e.m. of three independent experiments. Statistical significance was determined by one-way ANOVA for **b**, **c**, and **e**. Experiments for **a**, **b**, and **d** were repeated three times independently with similar results and representative images/blots are shown.

Supplementary Fig. 2

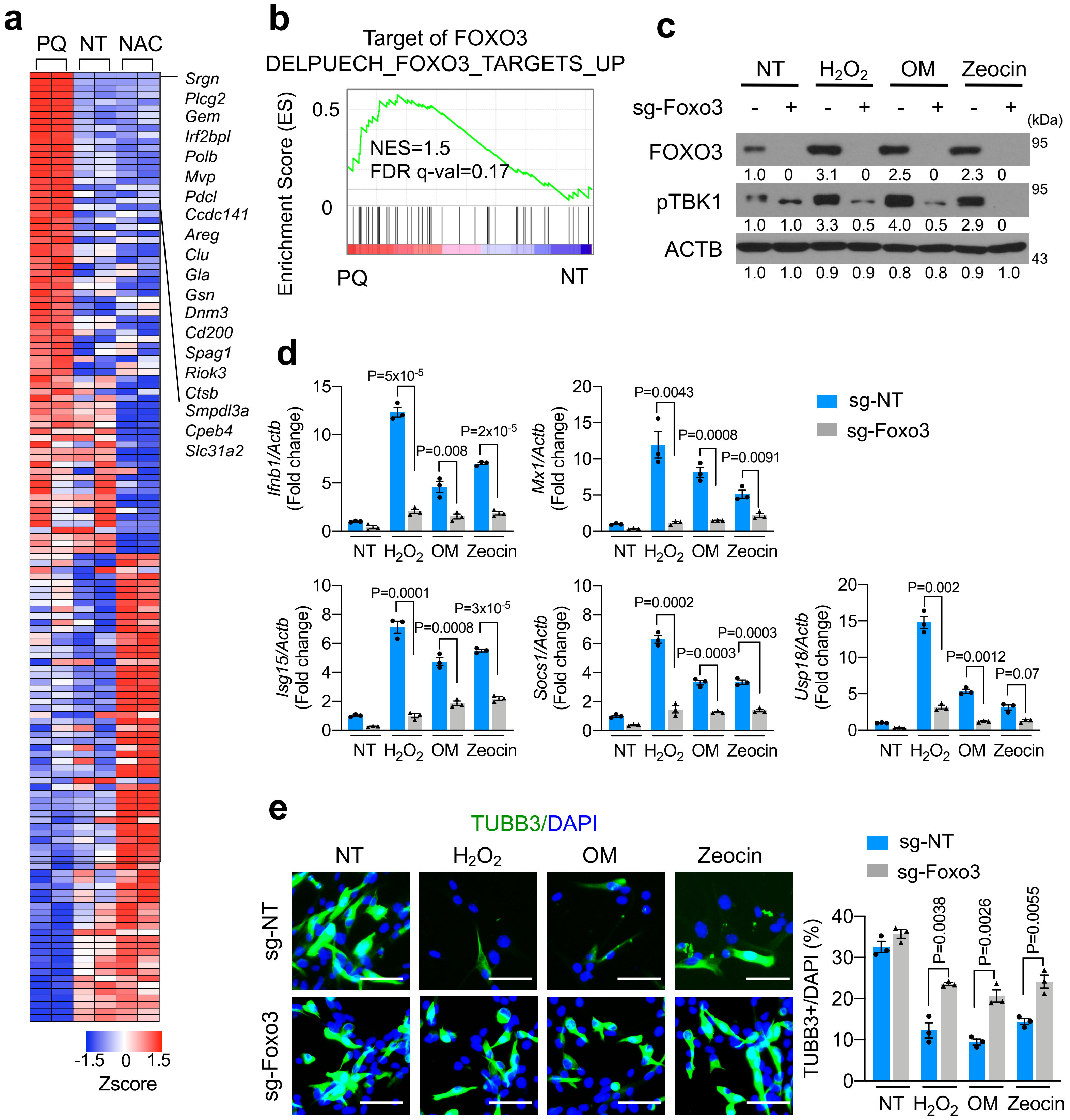

Supplementary figure 2. Cellular stress-activated FOXO3 is necessary for IFN-I signaling and suppresses neuronal differentiation. a. Heat map visualization of FOXO3 activation related gene expressions from NSPCs treated with PQ or NAC as in Fig. 1d-e. Top of 20 genes are presented on the right. The complete list of genes is included as source data. **b.** One-sided GSEA analysis of PQ-upregulated genes showed significant enrichment of 'DELPUECH_FOXO3_TARGETS_UP' gene set. **c.** Representative WB results for indicated proteins in NSPCs expressing sg-Foxo3 or sg-NT following 48h treatment of H_2O_2 (200 μM), oligomycin A (OM, 2.5 μM), or zeocin (100 μg/ml). **d.** qRT-PCR results of ISGs following 4 days of H_2O_2 (200 μM), oligomycin (OM, 2.5 μM), or zeocin (100 μg/ml) treatment. Mean ± s.e.m. of three independent experiments. **e.** Representative IF images of TUBB3 on 3 day of differentiation following 48h indicated treatments. Scale bar = 50 μm. Right, % of TUBB3-positive cells is plotted. Mean ± s.e.m. of three independent experiments. Statistical significance was determined by one-way ANOVA for **d** and **e.** Experiments for **c** and **e** were repeated three times independently with similar results and representative images/blots are shown.

Supplementary Fig. 3

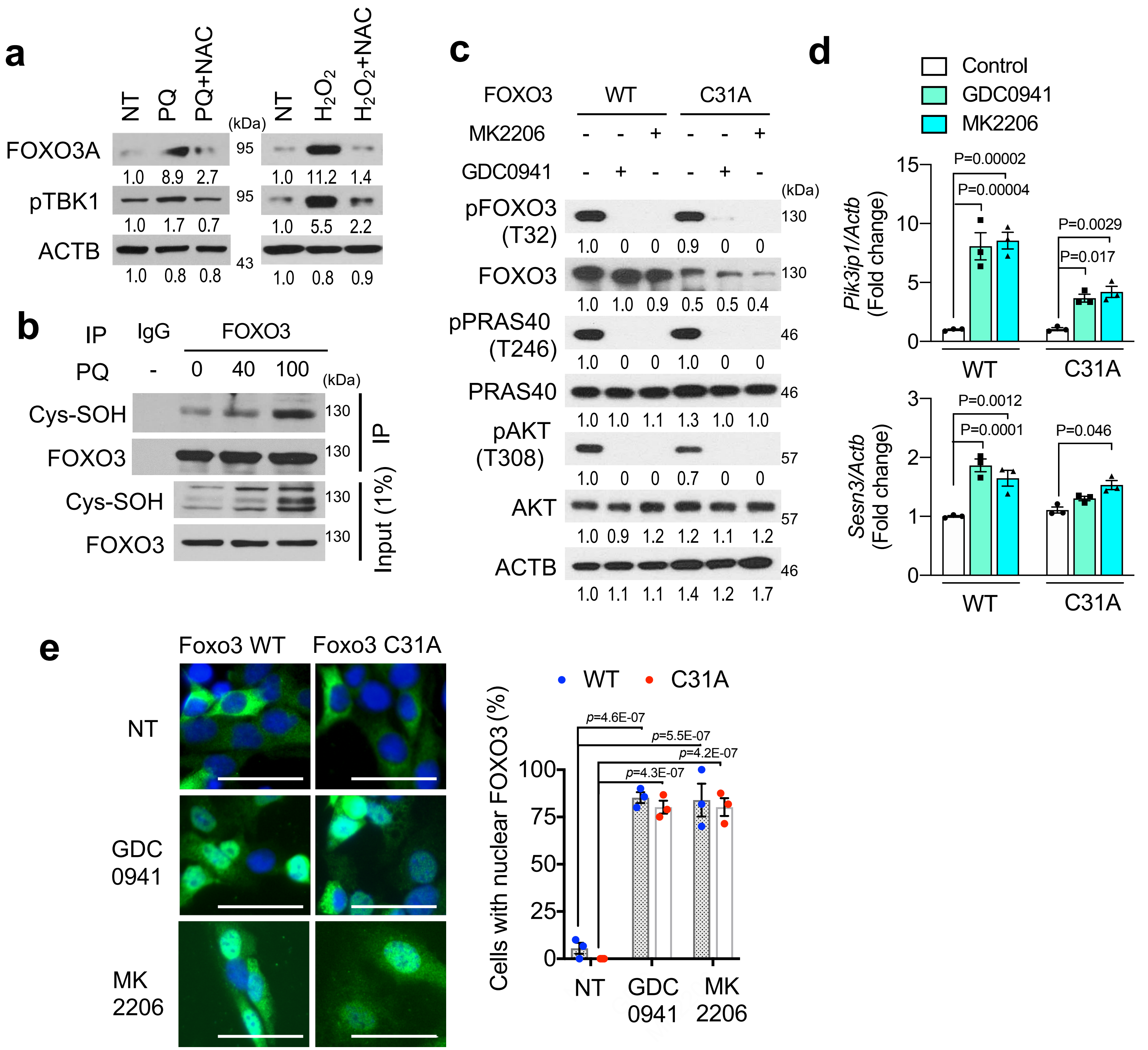

Supplementary figure 3. FOXO3 C31A retains its nuclear localization under PI3K/AKT inhibition. a. Representative WB results of indicated proteins following 24h treatments of PQ (5 µM), H_2O_2 (200 µM) or NAC (5 mM). **b**. WB results of Cysteine sulfenylation following immunoprecipitation of FOXO3. **c.** WB results of Foxo3 WT or Foxo3 C31A mutant expressing NSPCs. MK2206 (2 µM) or GDC0941 (500 nM) were treated for 0.5 hr. **d**. qRT-PCR results for transcriptional targets of FOXO3. Mean ± s.e.m. of three independent experiments. **e**. Microscopic analysis of FOXO3-EGFP following the same treatment as (c). Scale bar= 10 µm. % of cells with nuclear FOXO3 is plotted on the right. Mean ± s.e.m. of three independent experiments. Statistical significance was determined by one-way ANOVA for **d** and **e**. Mean ± s.e.m. of three independent experiments. Experiments for **a, b, c**, and **e** were repeated three times independently with similar results and representative images/blots are shown.

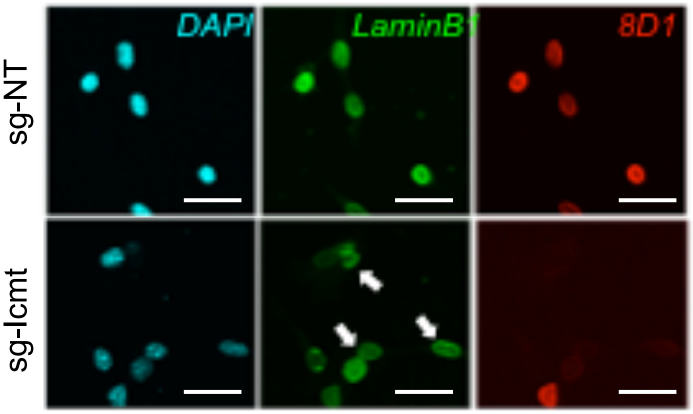

Supplementary figure 4. Depletion of ICMT inhibits prelamin B1 processing. IF for ICMT depleted NSPC (sg-Icmt) cultures in comparison to control NSPC (sg-NT). Arrows point to nuclei with lamin B1 expression but lacking 8D1 immunoreactivity. Scale bar= 20 µm. Experiment was repeated three times with similar results and representative images are shown.

Supplementary Fig. 5

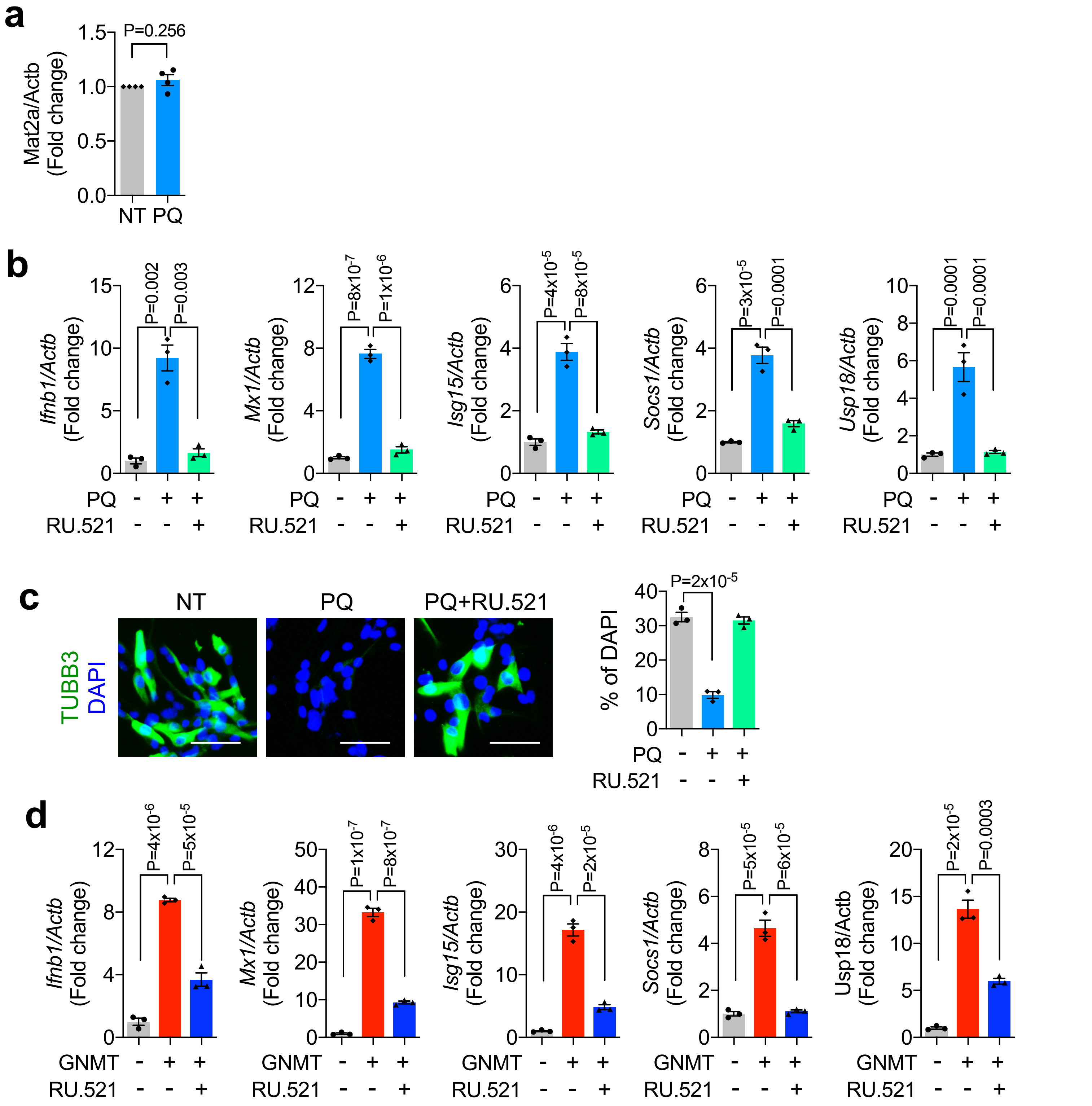

Supplementary figure 5. cGAS inhibition attenuates IFN-I response and offsets ROS inhibition of neuronal differentiation. a. qRT-PCR result of Mat2a in NSPCs following 2 days of PQ (10 μM) treatment. Mean ± s.e.m. of 4 independent experiments. **b.** qRT-PCR results of ISGs following 4 days of PQ (10 μM) with or without RU.521 (2 μM) treatment. Mean ± s.e.m. of three independent experiments. **c.** Representative IF images of TUBB3 on 3 day of differentiation. Scale bar= 50 μm. % of TUBB3-positive cells is plotted on the right. Mean ± s.e.m. of three independent experiments. **d.** qRT-PCR results of ISGs on day 4 of GNMT induction with or without RU.521 treatment. Mean ± s.e.m. of three independent experiments. Statistical significance was determined by two-sided unpaired t-test for **a** and by one-way ANOVA for **b-d**. Experiment for **c** was repeated three times independently with similar results and representative images are shown.

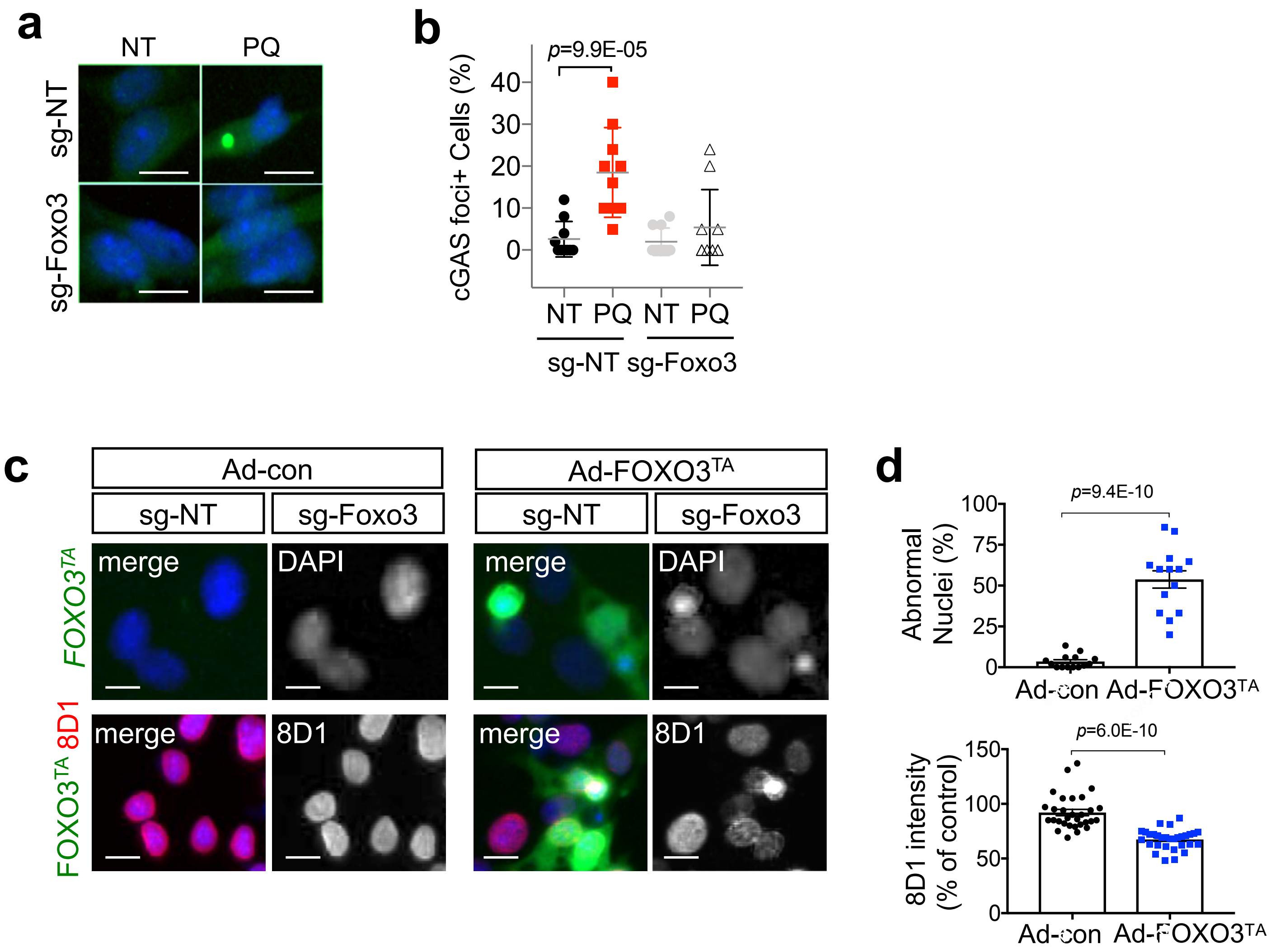

Supplementary figure 6. Stress induced-FOXO3 activates IFN-I response and interferes with lamin processing. a. Microscopic analysis for cGAS-GFP reporter following 1 day of PQ treatment. sg-NT: non-targeted guide RNA, sg-Foxo3: Foxo3 targeted guide RNA. Scale bar= 4 μm. **b**. Quantitation of the percent of cells with cGAS-GFP foci in **a** images. Mean ± s.e.m. of ten images. **c.** IF for lamin processing (8D1) on either adenovirus for control or FOXO3^{TA} infected NSPC. Scale bar= 4 μm. **d.** Quantitation of the percent of cells with abnormal nuclei and mature lamin B1 (8D1) positive nuclei. Mean ± s.e.m. of thirteen (upper) and fourteen (lower) images. Statistical significance was determined one-way ANOVA for **b** and by two-sided unpaired t-test for **d**. Experiment for **a** and **c** were repeated three times independently with similar results and representative images are shown.

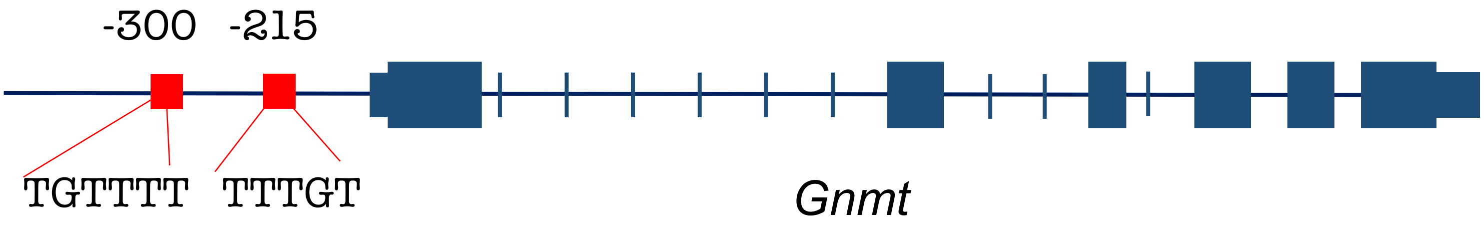

Supplementary figure 7. FOXO3 binding sites on *Gnmt* locus. The diagram represents two putative FOXO DBE on mouse Gnmt locus.

Supplementary Fig. 8

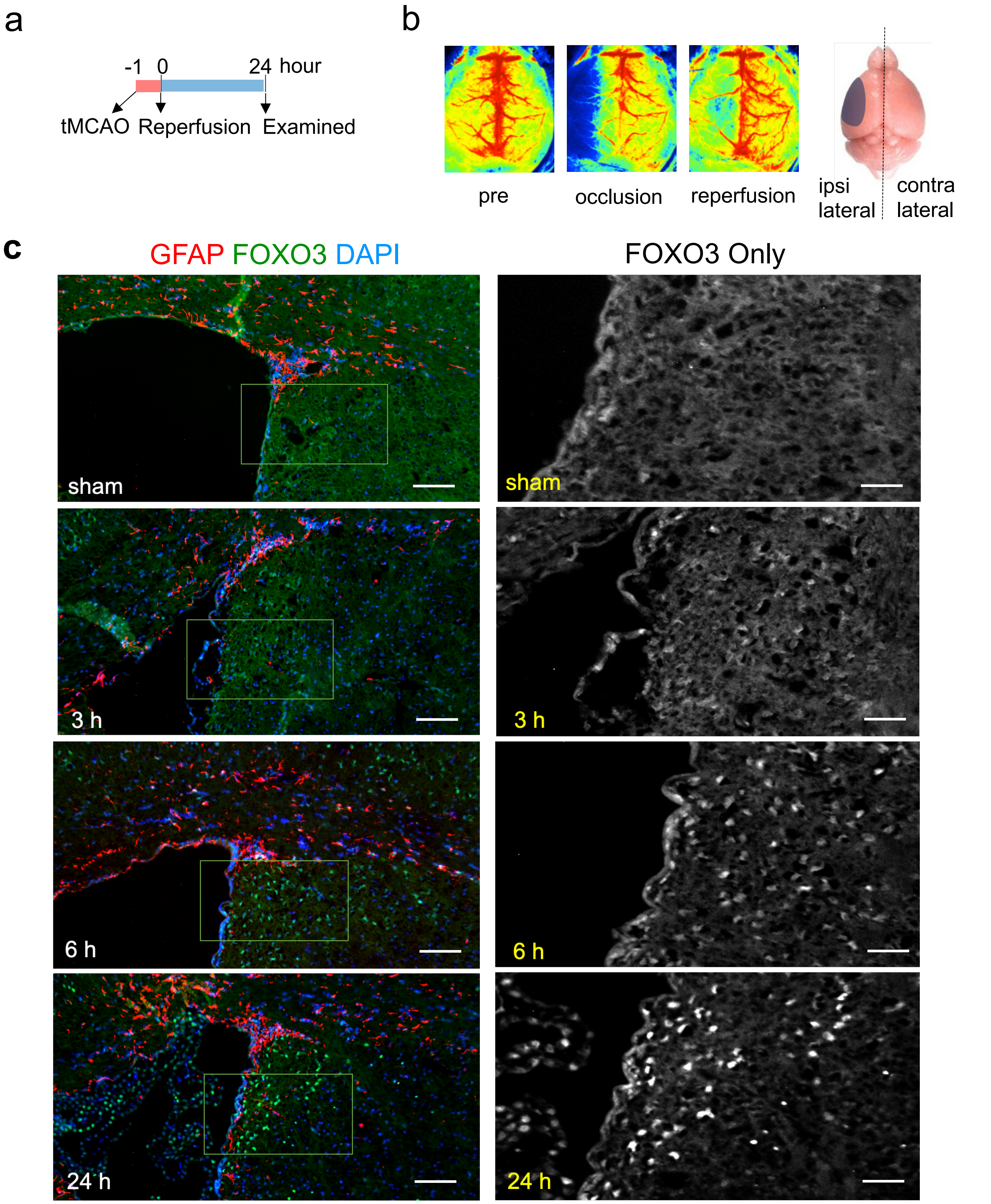

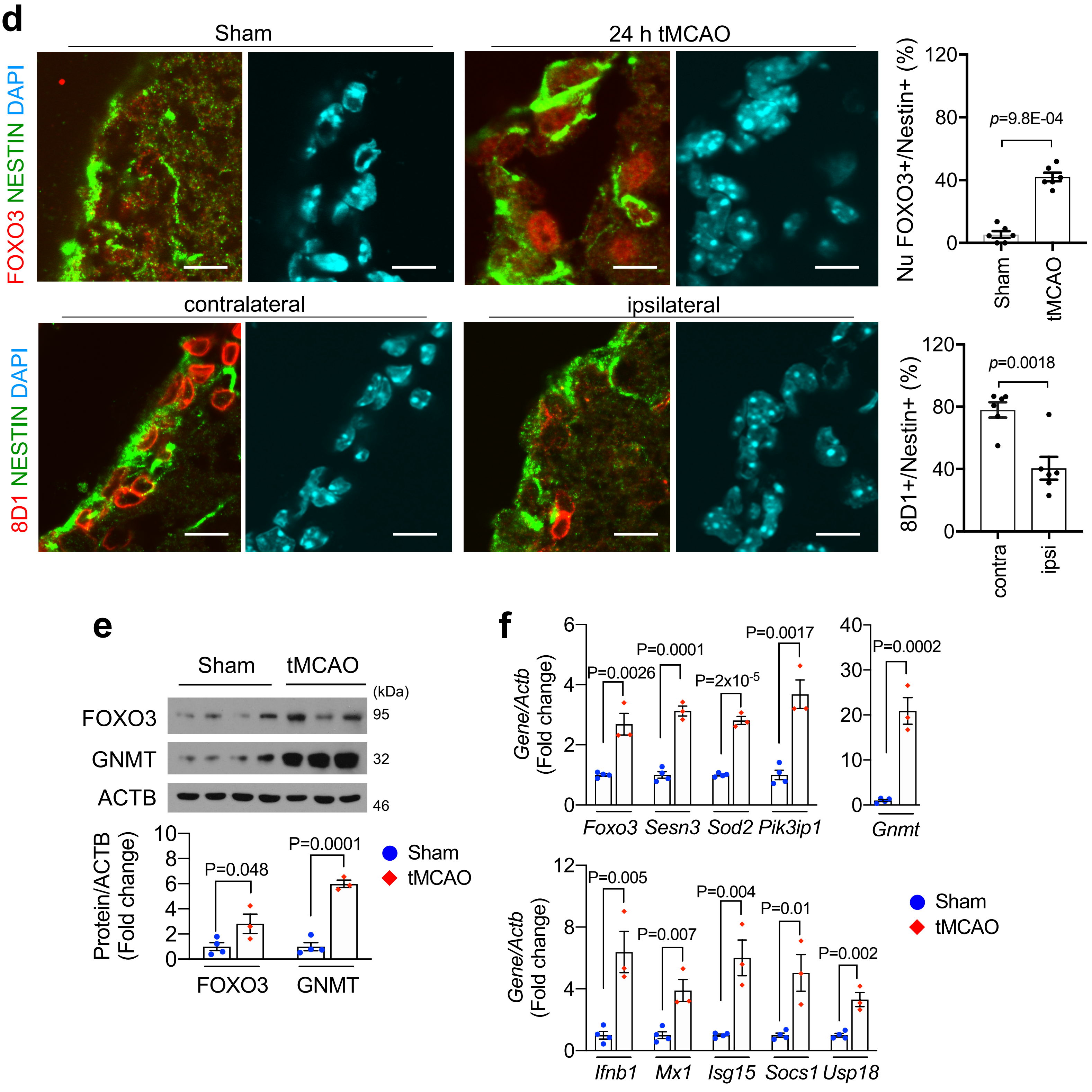
d
Sham
24 h tMCAO
FOXO3 NESTIN DAPI
Nu FOXO3+/Nestin+ (%)
p=9.8E-04
Sham
tMCAO
contralateral
ipsilateral
8D1 NESTIN DAPI
8D1+/Nestin+ (%)
p=0.0018
contra
ipsi
e
Sham
tMCAO
(kDa)
FOXO3
95
GNMT
32
ACTB
46
Protein/ACTB (Fold change)
P=0.048
P=0.0001
FOXO3
GNMT
Sham
tMCAO
f
Gene/Actb (Fold change)
P=0.0026
P=0.0001
P=2x10-5
P=0.0017
P=0.0002
Foxo3
Sesn3
Sod2
Pik3ip1
Gnmt
P=0.005
P=0.007
P=0.004
P=0.01
P=0.002
Ifnb1
Mx1
Isg15
Socs1
Usp18
Sham
tMCAO

Supplementary figure 8. **tMCAO model. a.** Schema for the timeline of analysis. **b.** Doppler analysis for brains before, during, and after MCAO. **c.** Representative co-IF results for FOXO3 and GFAP on time course analysis of tMCAO brain sections on the ipsilateral side SVZ. Scale bar= 100 μm. FOXO3 staining within each boxed region is shown on the right. **d.** Representative co-IF results for FOXO3 or mature lamin B1 (8D1) marked with Nestin in SVZ. Scale bar= 10 μm. Right, quantitation of multiple sections was plotted. Mean ± s.e.m. of six multiple sections from three experimental animals. **e.** Upper, WB results of indicated proteins in the brain. Lower, quantitation of band intensity was plotted. Mean ± s.e.m. of four (Sham) or three (tMCAO) experimental animals. **f.** qRT-PCR results of FOXO3 transcriptional targets (upper) and ISGs (lower) in the brain. Mean ± s.e.m. of four (Sham) or three (tMCAO) experimental animals. About 1 mm^2 around SVZ was dissected out for the analysis of protein and mRNA expressions. Statistical significance was determined by two-sided unpaired t-test for **d**-**f**. Experiment for **c-e** were repeated three times independently with similar results and representative images/blots are shown.

a

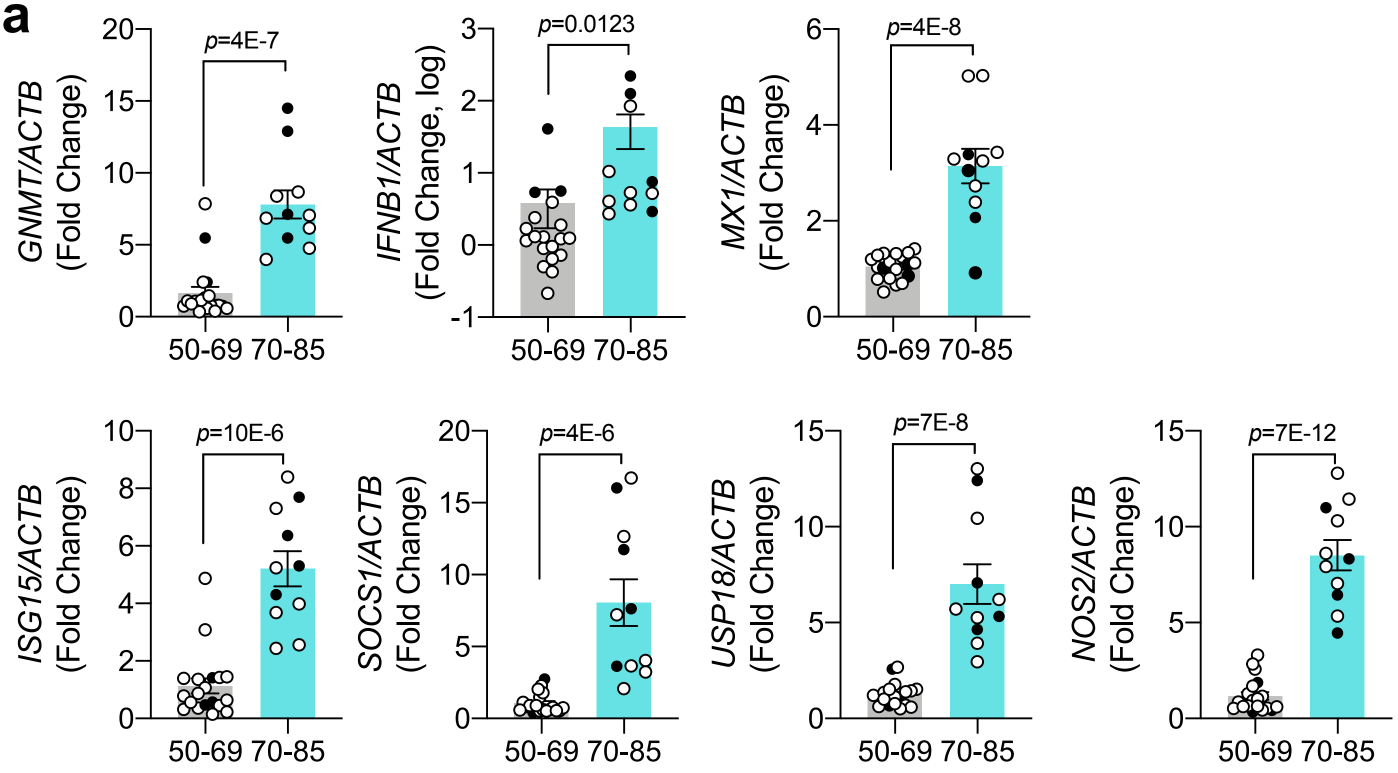

b

AGE	SEX	PMI	AGE	SEX	PMI	AGE	SEX	PMI
52	Male	19.55	60	Male	27.82	70	Female	17.18
52	Male	17.07	61	Male	26.9	71	Male	23.5
53	Male	23.57	63	Female	23.58	72	Male	18.85
53	Male	24.17	63	Male	20.58	76	Male	18.25
53	Male	19.22	66	Male	20.85	76	Female	8.08
53	Male	15.98	66	Female	23.2	77	Male	14.55
54	Male	19.9	68	Male	24.75	79	Male	22.93
55	Male	16.43	69	Male	31.25	80	Male	18.3
55	Male	17.8				83	Female	18.5
58	Male	20.47				84	Male	28.77
60	Female	28.15				84	Female	22.65

Supplementary Figure 9. Activated IFN-I response in human aged brains. a. qRT-PCR measurement of ISGs and GNMT in cerebellum samples from aging human cohort. Open and closed dots correspond to male and female, respectively. Mean ± s.e.m of nineteen (50-69) or eleven (70-85) human brain samples. Statistical significance was determined by two-sided unpaired t-test. **b.** Summary of human subjects' information. PMI: Postmortem interval. Our analysis is the extension of already procured and deidentified brain samples from human age groups from the branch of NIH NeuroBioBank (Harvard Brain Tissue Resource Center is supported by PHS contract, HHSN-271-2013-00030C). This study is, therefore, not subject to the separate approval of the study protocol.
