# Supplementary material for: Cellular stress signaling activates type-I IFN response through FOXO3-regulated lamin posttranslational modification
Source: Nat Commun. 2021 Jan 28;12:640. doi: 10.1038/s41467-020-20839-0 (PMC7843645; doi:10.1038/s41467-020-20839-0)
Supplement: Supplementary file 3 — Reporting Summary [file 41467_2020_20839_MOESM3_ESM.pdf]

nature research

Corresponding author(s): Jihye Paik

Last updated by author(s): Nov 7, 2020

Reporting Summary

Nature Research wishes to improve the reproducibility of the work that we publish. This form provides structure for consistency and transparency in reporting. For further information on Nature Research policies, see our Editorial Policies and the Editorial Policy Checklist.

Statistics

For all statistical analyses, confirm that the following items are present in the figure legend, table legend, main text, or Methods section.

n/a	Confirmed	
☐	☒	The exact sample size (*n*) for each experimental group/condition, given as a discrete number and unit of measurement
☐	☒	A statement on whether measurements were taken from distinct samples or whether the same sample was measured repeatedly
☐	☒	The statistical test(s) used AND whether they are one- or two-sided<br>*Only common tests should be described solely by name; describe more complex techniques in the Methods section.*
☒	☐	A description of all covariates tested
☐	☒	A description of any assumptions or corrections, such as tests of normality and adjustment for multiple comparisons
☐	☒	A full description of the statistical parameters including central tendency (e.g. means) or other basic estimates (e.g. regression coefficient) AND variation (e.g. standard deviation) or associated estimates of uncertainty (e.g. confidence intervals)
☐	☒	For null hypothesis testing, the test statistic (e.g. *F*, *t*, *r*) with confidence intervals, effect sizes, degrees of freedom and *P* value noted<br>*Give P values as exact values whenever suitable.*
☒	☐	For Bayesian analysis, information on the choice of priors and Markov chain Monte Carlo settings
☒	☐	For hierarchical and complex designs, identification of the appropriate level for tests and full reporting of outcomes
☒	☐	Estimates of effect sizes (e.g. Cohen's *d*, Pearson's *r*), indicating how they were calculated

Our web collection on statistics for biologists contains articles on many of the points above.

Software and code

Policy information about availability of computer code

Data collection	qRT-PCR data were collected by ABI7500 fast software. Microscopic fluorescent image data were collected by EVOS FL Auto Software Revision 1.7 Software and FLUOVIEW software. RNA sequencing data were collected by HiSeq Control Software version 3.3.76.
Data analysis	Image data and western blot data were analyzed by ImageJ 1.52k. RNA-seq data were aligned to the mm9 reference genome using TopHat, and Cufflinks was used to measure transcript abundances. GSEA software was used for the gene expression analysis. GraphPad Prism software (version 7.0e) was used for all statistical analysis.

For manuscripts utilizing custom algorithms or software that are central to the research but not yet described in published literature, software must be made available to editors and reviewers. We strongly encourage code deposition in a community repository (e.g. GitHub). See the Nature Research guidelines for submitting code & software for further information.

Data

Policy information about availability of data

All manuscripts must include a data availability statement. This statement should provide the following information, where applicable:

- Accession codes, unique identifiers, or web links for publicly available datasets
- A list of figures that have associated raw data
- A description of any restrictions on data availability

The data that support the findings of this study are available within the article and its Supplementary Information files or from the corresponding author (J.P.) upon reasonable request. RNA-seq data can be found on Gene Expression Omnibus (GEO) database with accession number GSE146243. All the raw western blots and raw data are provided as a Source data file.

Field-specific reporting

Please select the one below that is the best fit for your research. If you are not sure, read the appropriate sections before making your selection.

☒ Life sciences ☐ Behavioural & social sciences ☐ Ecological, evolutionary & environmental sciences

For a reference copy of the document with all sections, see nature.com/documents/nr-reporting-summary-flat.pdf

Life sciences study design

All studies must disclose on these points even when the disclosure is negative.

Sample size	For in vitro studies, cultured cells generally have little variability and the coefficient of variation (CV) is often <0.1. Under this assumption, there will be 95% power to detect a 1.5-fold-change with a 2-sided alpha of 0.05 using a t-test and 3 biological replicates. With this a minimum sample size of 3 was used for all the in vitro experiments. A sample size of 3-4 for the in vivo MCAO experiments was chosen based on the previous study (doi.org/10.1038/ncomms8893). 30 human postmortem tissue samples were analyzed based on sample availability. The data generated from RNA-seq follow a negative binomial distribution. Assuming such a distribution and a CV of 0.1 for cultured cells, 3 biological replicates with 30× sequencing coverage will have at least 80% power to detect a 1.75-fold-change.
Data exclusions	No data were excluded from the analyses of samples.
Replication	Western blot, qRT-PCR, immunofluorescence, and metabolite mass analysis were replicated three times. RNA-sequencing was replicated twice. All experiments were successfully replicated and representative data were shown.
Randomization	Mice from the same strain were randomly allocated to different groups (sham or tMCAO).
Blinding	In data collection and analysis (e.g., qRT-PCR, RNA-seq, western blot, Immunofluorescence), the performer(s) was blinded with experimental design. For in vivo experiment, formal blinding was not performed as we have a single surgeon to perform the tMCAO. However, following sample procurement and processing/analysis was performed by a different researcher who is blinded to the treatment assignment.

Reporting for specific materials, systems and methods

We require information from authors about some types of materials, experimental systems and methods used in many studies. Here, indicate whether each material, system or method listed is relevant to your study. If you are not sure if a list item applies to your research, read the appropriate section before selecting a response.

Materials & experimental systems

n/a	Involved in the study	
☐	☒	Antibodies
☐	☒	Eukaryotic cell lines
☒	☐	Palaeontology and archaeology
☐	☒	Animals and other organisms
☐	☒	Human research participants
☒	☐	Clinical data
☒	☐	Dual use research of concern

Methods

n/a	Involved in the study	
☒	☐	ChIP-seq
☒	☐	Flow cytometry
☒	☐	MRI-based neuroimaging

Antibodies

Antibodies used

Rabbit anti-ICMT AntibodyPlus Cat# A10293 WB (1:2,000)
Rabbit anti-H3K4me3 Abcam Cat# ab8580 WB (1:10,000), IF (1:10,000)
Rabbit anti-Lamin B1 Abcam Cat# 133741 WB (1:5,000), IF (1:10,000)
Mouse anti-βIII-Tubulin Abcam Cat# ab78078 WB (1:5,000), IF (1:300)
Chicken anti-Nestin Abcam Cat# ab134017 IF (1:100)
Rabbit anti-DCX Cell signaling technology Cat# 4604 IF (1:100)
Mouse anti-Histone H3 Cell signaling technology Cat# 14269 WB (1:10,000)
Rabbit anti-Akt Cell signaling technology Cat# 4691 WB (1:5,000)
Rabbit anti-pAkt (S473) Cell signaling technology Cat# 9271 WB (1:1,000)
Rabbit anti-FOXO3 Cell signaling technology Cat# 12829 WB (1:1,000), IF (1:100)
Rabbit anti-pFOXO3 Cell signaling technology Cat# 9466 WB (1:1,000)
Rabbit anti-pSTAT1 (Y701) Cell signaling technology Cat# 9167 WB (1:1,000)
Rabbit anti-pTBK1 Cell signaling technology Cat# 5483 WB (1:1,000)
Rabbit anti-pPRAS40 (T246) Cell signaling technology Cat# 2997 WB (1:2,000)
Rabbit anti-PRAS40 Cell signaling technology Cat# 2691 WB (1:2,000)
Rabbit anti-PDGFRA Cell signaling technology Cat# 3174 IF (1:100)

Mouse anti-β-actin Millipore Sigma Cat# A3853 WB (1:10,000)
Mouse anti-FLAG Millipore Sigma Cat# F3165 WB (1:1,000)
Mouse anti-Nestin Millipore Sigma Cat# MAB353 IF (1:300)
Rabbit anti-Cys Sulfenic acid Millipore Sigma Cat# ABS30 WB (1:1,000)
Rat anti-IFNAR2 Novus Biologicals Cat# MAB1083 WB (1:1,000)
Mouse anti-GFAP OriGene Technologies Cat# TA336707 IF (1:100)
Rabbit anti-GFAP Aglient DAKO Cat# Z0334 IF (1:500)
anti-GNMT SantaCruz Cat# sc68871 WB (1:500)
anti-Lamin B1 (8D1) SantaCruz Cat# sc56144 WB (1:1,000), IF (1:100)
Rabbit anti-FOXO3 Homemade ChIP (10 μg/mL)
Alexa 488 anti-mouse ThermoFisher Scientific Cat# A21202 IF (1:1,000)
Alexa 488 anti-rabbit ThermoFisher Scientific Cat# A21206 IF (1:1,000)
Alexa 488 anti-chicken ThermoFisher Scientific Cat# A11039 IF (1:1,000)
Alexa 594 anti-mouse ThermoFisher Scientific Cat# A21203 IF (1:1,000)
Alexa 594 anti-rabbit ThermoFisher Scientific Cat# A21207 IF (1:1,000)
Goat anti-mouse IgG HRP ThermoFisher Scientific Cat# 31430 WB (1:5,000)
Goat anti-rabbit IgG HRP ThermoFisher Scientific Cat# 31460 WB (1:5,000)
Goat anti-rat IgG HRP ThermoFisher Scientific Cat# 31470 WB (1:5,000)
Pierce™ Anti-DYKDDDDK Magnetic Agarose ThermoFisher Scientific Cat# A36797 IP (10 μl/500 mg proteins)

Validation

anti-ICMT (https://antibodyplus.com/primary-antibody/rabbit-polyclonal-icmt-antibody-a10293/),
Rabbit anti-H3K4me3 (https://www.abcam.com/histone-h3-tri-methyl-k4-antibody-chip-grade-ab8580.html)
Rabbit anti-Lamin B1 (https://www.abcam.com/lamin-b1-antibody-epr8985b-ab133741.html)
Mouse anti-βIII-Tubulin (https://www.abcam.com/beta-iii-tubulin-antibody-2g10-neuronal-marker-ab78078.html)
Chicken anti-Nestin (https://www.abcam.com/nestin-antibody-ab134017.html)
Rabbit anti-DCX (https://www.cellsignal.com/products/primary-antibodies/doublecortin-antibody/4604)
Mouse anti-Histone H3 (https://www.cellsignal.com/products/primary-antibodies/histone-h3-1b1b2-mouse-mab/14269)
Rabbit anti-Akt(https://www.cellsignal.com/products/primary-antibodies/akt-pan-c67e7-rabbit-mab/4691)
Rabbit anti-pAkt (S473) (https://www.cellsignal.com/products/primary-antibodies/phospho-akt-ser473-antibody/9271)
Rabbit anti-FOXO3 (https://www.cellsignal.com/products/primary-antibodies/foxo3a-d19a7-rabbit-mab/12829)
Rabbit anti-pFOXO3 (https://www.cellsignal.com/products/primary-antibodies/phospho-foxo3a-ser253-antibody/9466)
Rabbit anti-pSTAT1 (Y701) (https://www.cellsignal.com/products/primary-antibodies/phospho-stat1-tyr701-58d6-rabbit-mab/9167)
Rabbit anti-pTBK1 (https://www.cellsignal.com/products/primary-antibodies/phospho-tbk1-nak-ser172-d52c2-xp-rabbit-mab/5483)
Rabbit anti-pPRAS40 (T246) (https://www.cellsignal.com/products/primary-antibodies/phospho-pras40-thr246-c77d7-rabbit-mab/2997)
Rabbit anti-PRAS40 (https://www.cellsignal.com/products/primary-antibodies/pras40-d23c7-xp-rabbit-mab/2691)
Rabbit anti-PDGFRA (https://www.cellsignal.https://www.sigmaaldrich.com/catalog/product/mm/mab353?lang=en&region=UScom/products/primary-antibodies/pdgf-receptor-a-d1e1e-xp-rabbit-mab/3174)
Mouse anti-β-actin (https://www.sigmaaldrich.com/catalog/product/sigma/a3853?lang=en&region=US)
Mouse anti-FLAG (https://www.sigmaaldrich.com/catalog/product/sigma/f3165?lang=en&region=US)
Mouse anti-Nestin (https://www.sigmaaldrich.com/catalog/product/mm/mab353?lang=en&region=US)
Rabbit anti-Cys Sulfenic acid (https://www.sigmaaldrich.com/catalog/product/mm/abs30?lang=en&region=US)
Rat anti-IFNAR2 (https://www.novusbio.com/products/ifn-alpha-beta-r2-antibody-237526_mab1083)
Mouse anti-GFAP (https://www.origene.com/catalog/antibodies/primary-antibodies/ta336707/gfap-mouse-monoclonal-antibody-clone-id-5c10)
Rabbit anti-GFAP (https://www.agilent.com/en/product/immunohistochemistry/antibodies-controls/primary-antibodies/glial-fibrillary-acidic-protein-(dako-omnis)-76214)
anti-GNMT (https://www.scbt.com/p/gnmt-antibody-h-225)
anti-LaminB1(8D1) (https://www.scbt.com/p/lamin-b1-antibody-8d1?requestFrom=search)
Alexa probe secondary antibody (Alexa488 mouse: https://www.thermofisher.com/antibody/product/Donkey-anti-Mouse-IgG-H-L-Highly-Cross-Adsorbed-Secondary-Antibody-Polyclonal/A-21202, Alexa488 rabbit: https://www.thermofisher.com/antibody/product/Donkey-anti-Rabbit-IgG-H-L-Highly-Cross-Adsorbed-Secondary-Antibody-Polyclonal/A-21206, Alexa488 chicken: https://www.thermofisher.com/antibody/product/Goat-anti-Chicken-IgY-H-L-Secondary-Antibody-Polyclonal/A-11039, Alexa594 mouse: https://www.thermofisher.com/antibody/product/Donkey-anti-Mouse-IgG-H-L-Highly-Cross-Adsorbed-Secondary-Antibody-Polyclonal/A-21203, Alexa594 rabbit: https://www.thermofisher.com/antibody/product/Donkey-anti-Rabbit-IgG-H-L-Highly-Cross-Adsorbed-Secondary-Antibody-Polyclonal/A-21207)
HRP-conjugated secondary antibody (anti-mouse: https://www.thermofisher.com/antibody/product/Goat-anti-Mouse-IgG-H-L-Secondary-Antibody-Polyclonal/32430, anti-rabbit: https://www.thermofisher.com/antibody/product/Goat-anti-Rabbit-IgG-H-L-Secondary-Antibody-Polyclonal/31460, anti-rat: https://www.thermofisher.com/antibody/product/Goat-anti-Rat-IgG-H-L-Secondary-Antibody-Polyclonal/31470)
Pierce™ Anti-DYKDDDDK Magnetic Agarose (https://www.thermofisher.com/order/catalog/product/A36797#/A36797)
Our homemade rabbit anti-FOXO3 is working on human and mouse species and available for IF, WB, IHC, and ChIP. Specificity was validated on FOXO3 overexpressing and knockout cells.

Eukaryotic cell lines

Policy information about cell lines

Cell line source(s)	Primary murine NSPCs were isolated from subventricular zones (SVZ) of Ink/Arf null mice.<br>HEK293T cell line was purchased from ATCC (CRL-11268)
Authentication	We authenticated Ink/Arf null, FOXO null and p53 null primary cells by PCR genotyping of genomic DNA.<br>ATCC authenticated HEK293T.
Mycoplasma contamination	Cell line was tested and found to be mycoplasma free.
Commonly misidentified lines<br>(See ICLAC register)	No such line was used for the study

Animals and other organisms

Policy information about studies involving animals; ARRIVE guidelines recommended for reporting animal research

Laboratory animals	Mice were group housed (up to 5 per cage) in individually ventilated cages (Maxi-Miser, Thoren Caging Systems, Hazelton, PA) with ad libitum access to food (PicoLab Diet 5053, PMI, St Louis, MO) and acidified water (pH 2.5 to 2.8) in a temperature (22.2 ± 0.5 °C) and humidity (30-70%) controlled facility with 12:12-h light:dark cycle. The animal care and use program is accredited AAALAC. All animal experiments were approved by Weill Cornell Institutional Animal Care and Use Committee. 8-10 week-old C57BL/6J male mouse were used for this study.
Wild animals	The study does not include any wild animals.
Field-collected samples	The study does not include any such samples
Ethics oversight	All animal experiments were approved by Weill Cornell Institutional Animal Care and Use Committee.

Note that full information on the approval of the study protocol must also be provided in the manuscript.

Human research participants

Policy information about studies involving human research participants

Population characteristics	We attached the file that includes population characteristics (Human Research Characteristics.xls).
Recruitment	NA
Ethics oversight	Our analysis is the extension of already procured and deidentified brain samples from human age groups from the branch of NIH NeuroBioBank (Harvard Brain Tissue Resource Center is supported by PHS contract, HHSN-271-2013-00030C). This study is, therefore, not subject to the separate approval of the study protocol.

Note that full information on the approval of the study protocol must also be provided in the manuscript.
