# Supplementary material for: Cellular stress signaling activates type-I IFN response through FOXO3-regulated lamin posttranslational modification
Source: Nat Commun. 2021 Jan 28;12:640. doi: 10.1038/s41467-020-20839-0 (PMC7843645; doi:10.1038/s41467-020-20839-0)
Supplement: Supplementary file 4 — Description of Additional Supplementary Files [file 41467_2020_20839_MOESM4_ESM.pdf]

Description of Additional Supplementary Files

Supplementary Data 1. The list of GSEA analysis. PQ-upregulated, PQ-downregulated, NAC-upregulated, and NAC-downregulated one-sided GSEA results are listed.

Supplementary Data 2. The list of materials.
