# Supplementary material for: Cellular stress signaling activates type-I IFN response through FOXO3-regulated lamin posttranslational modification
Source: Nat Commun. 2021 Jan 28;12:640. doi: 10.1038/s41467-020-20839-0 (PMC7843645; doi:10.1038/s41467-020-20839-0)

Figure 1h

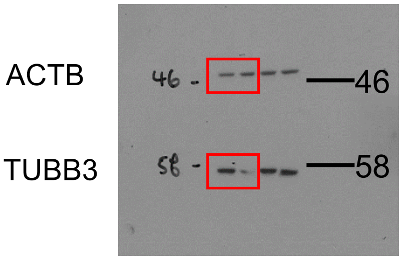

Figure 2a

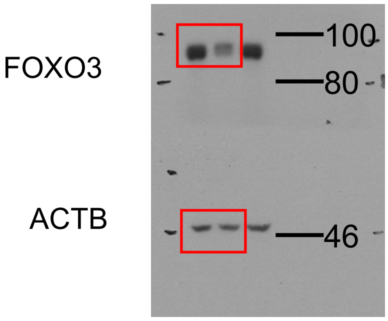

Figure 2b

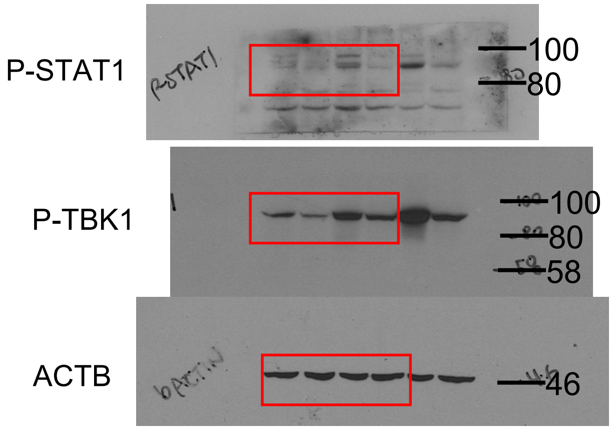

Figure 2f

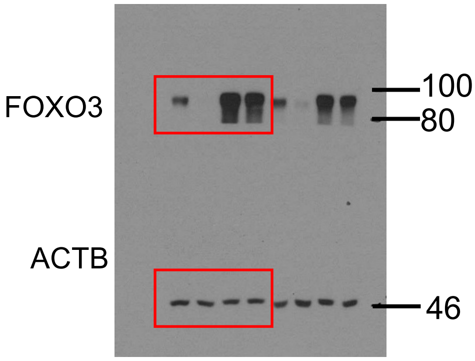

Figure 3c

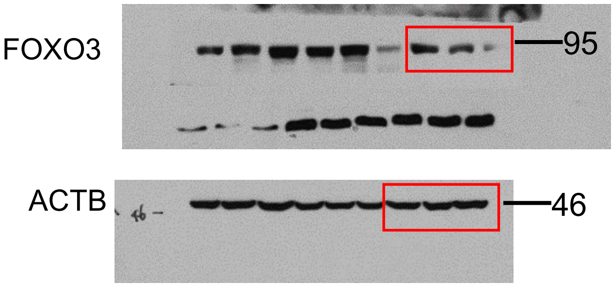

Figure 3d

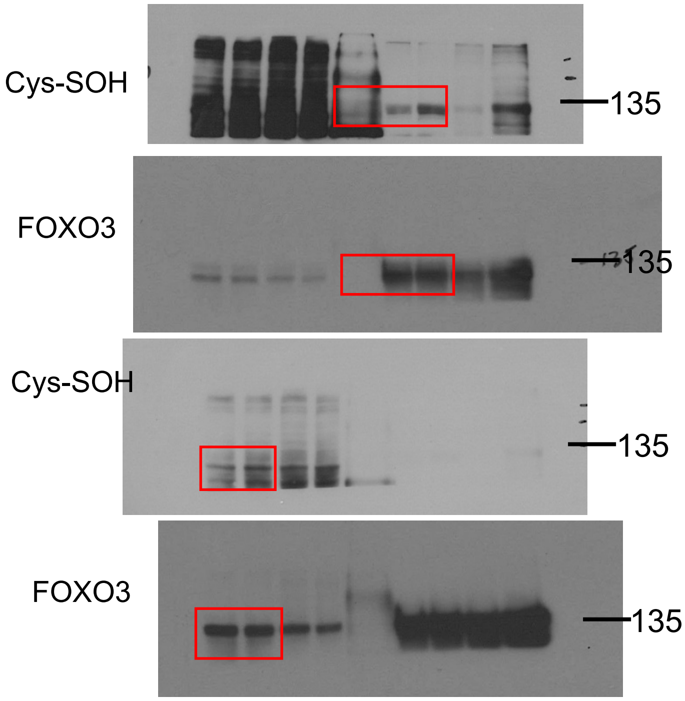

Figure 3f

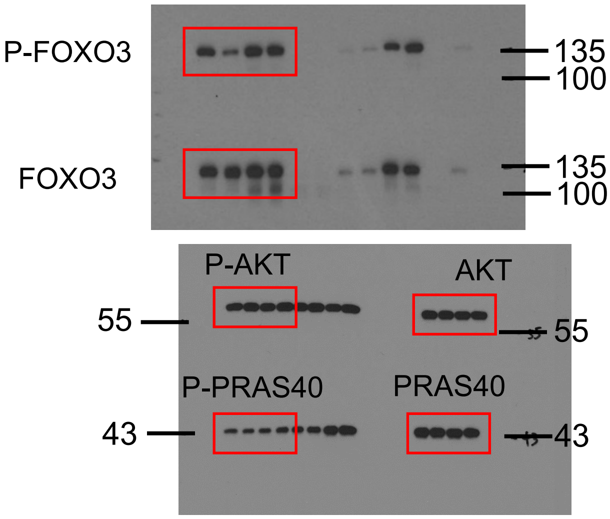

Figure 5d

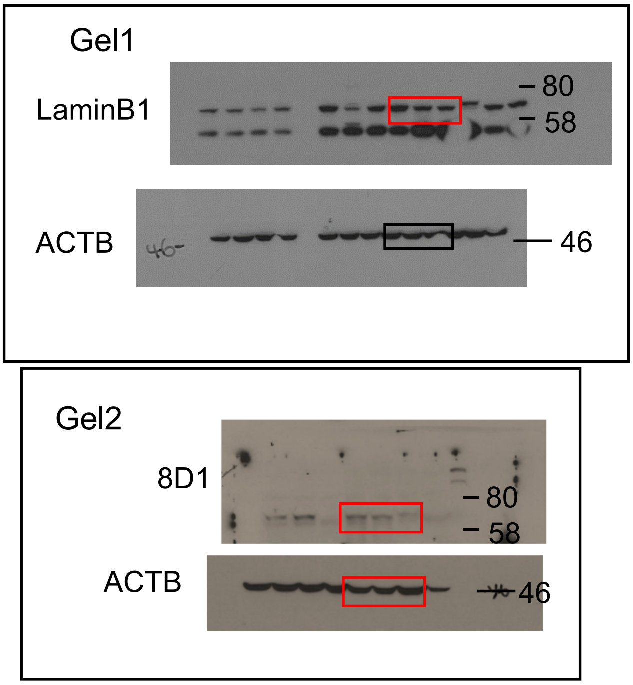

Figure 5g

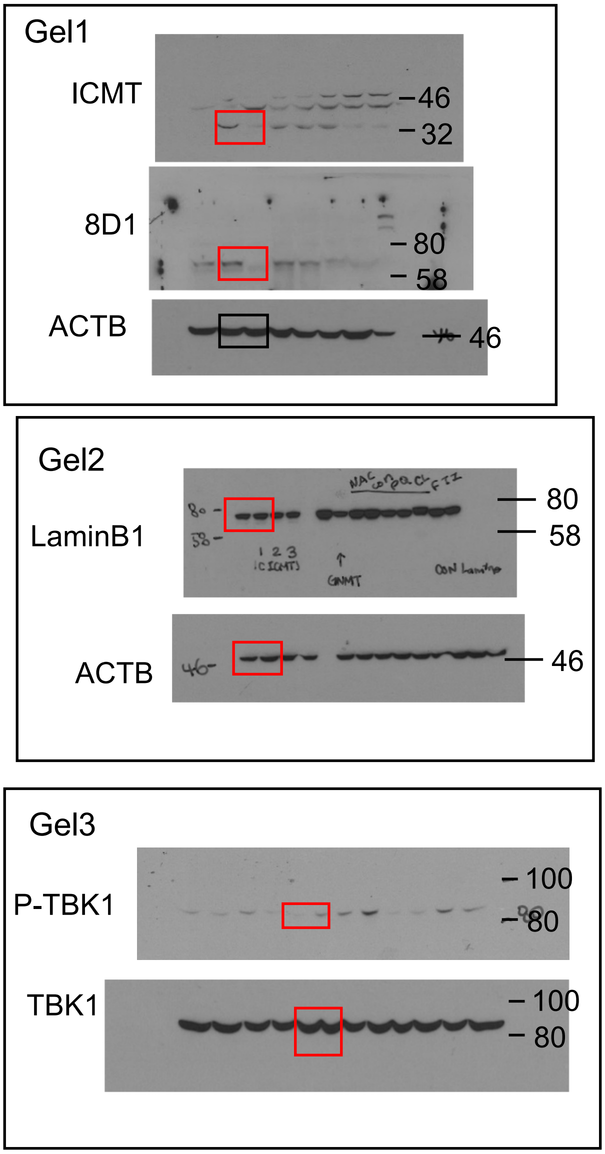

Figure 6b

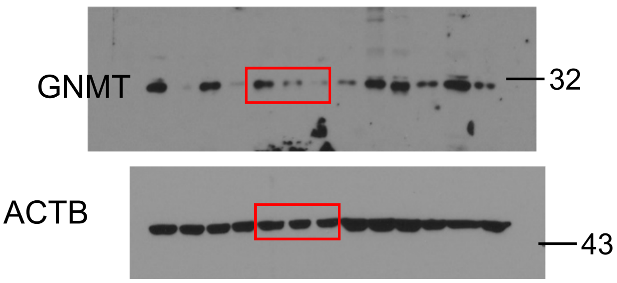

Figure 6d

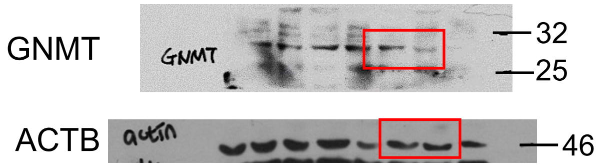

Figure 6f

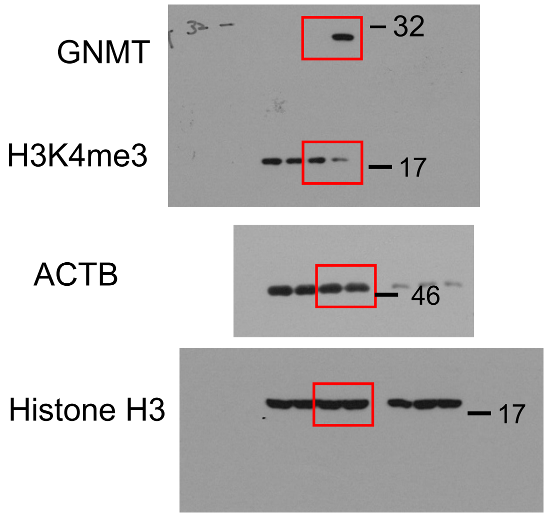

Figure 6j

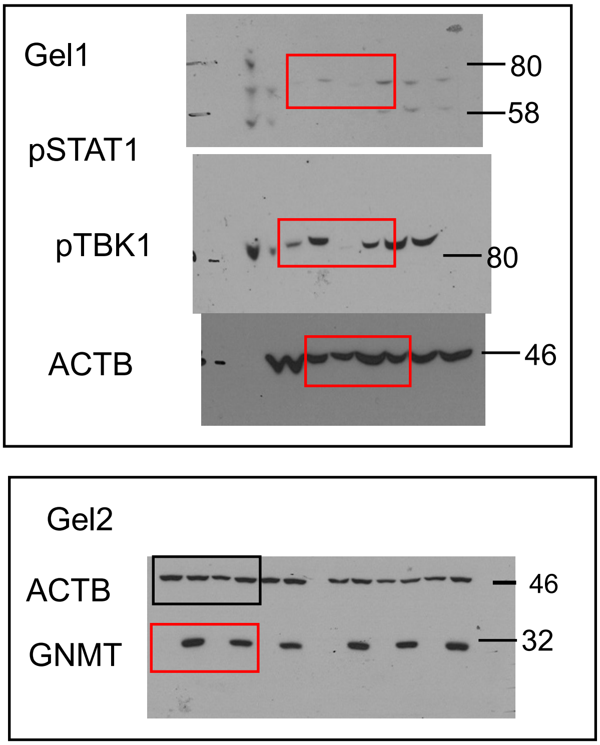

Figure 6l

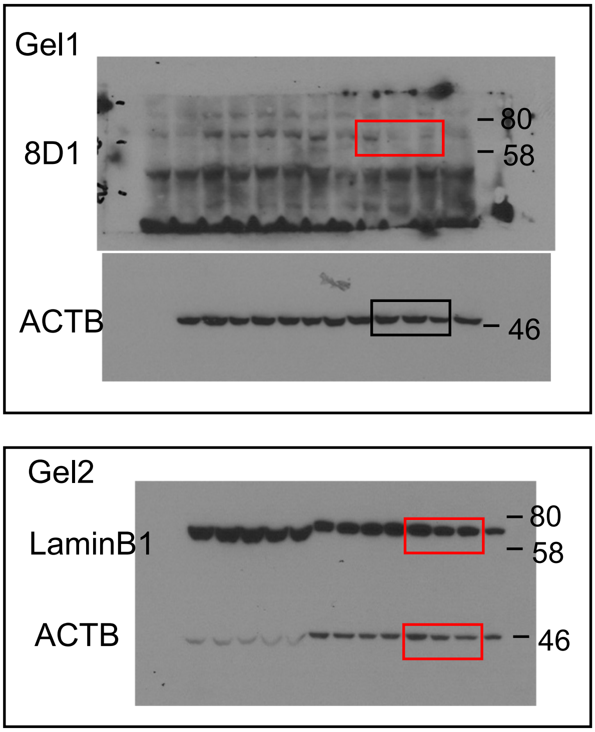

Figure 7d

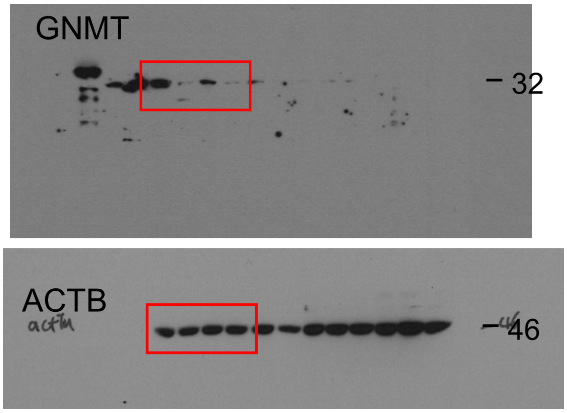

Figure 7i

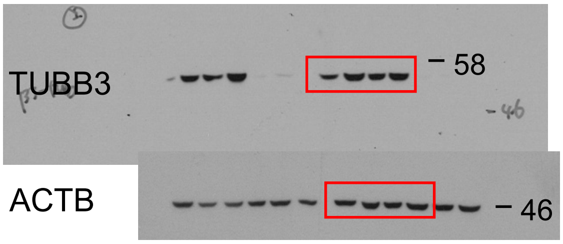

Figure 7g

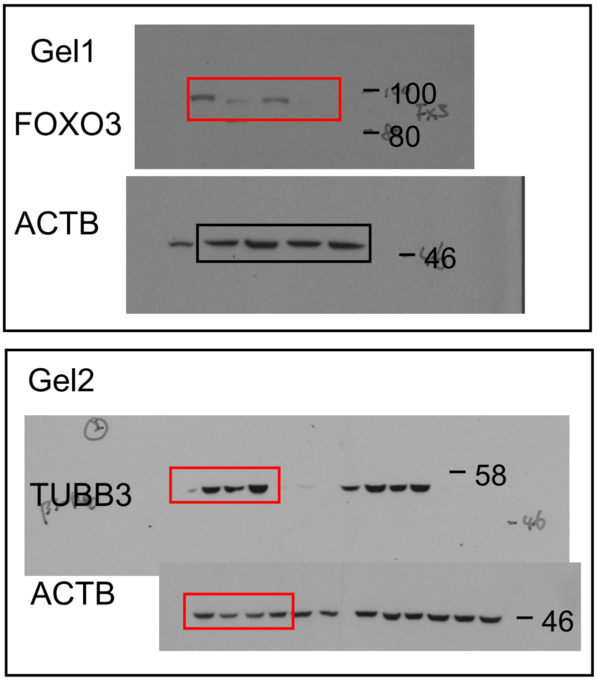

Figure 7k

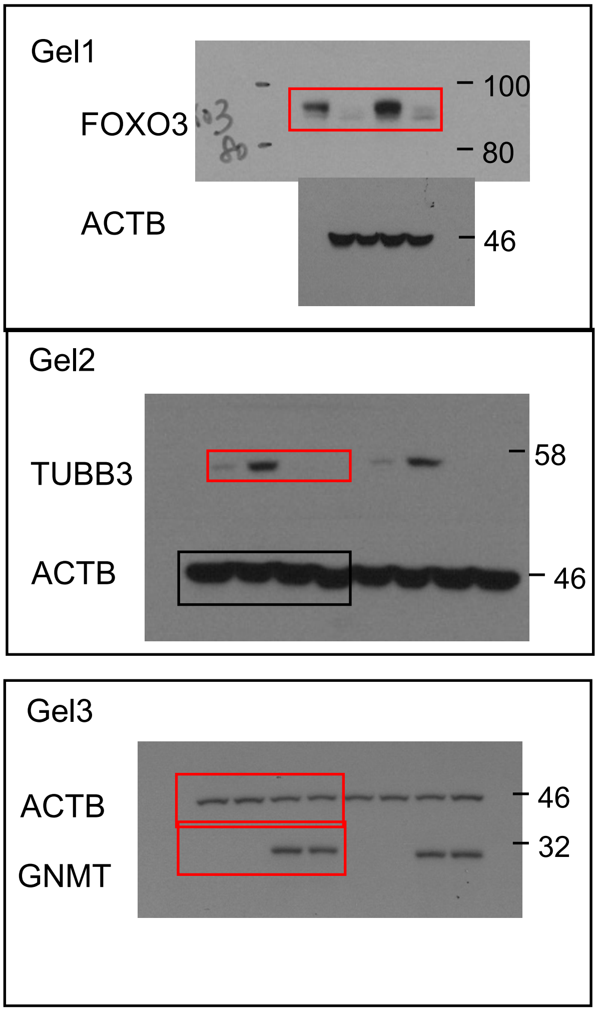

Supple Fig. 1a

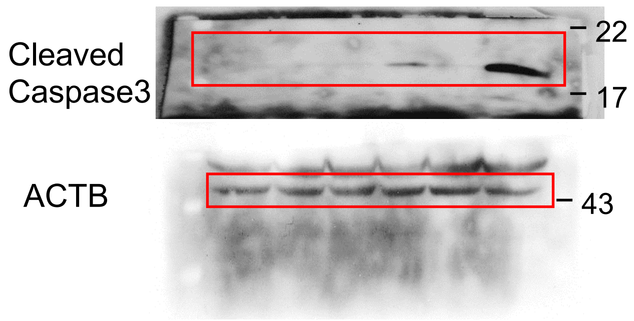

Supple Fig. 1d

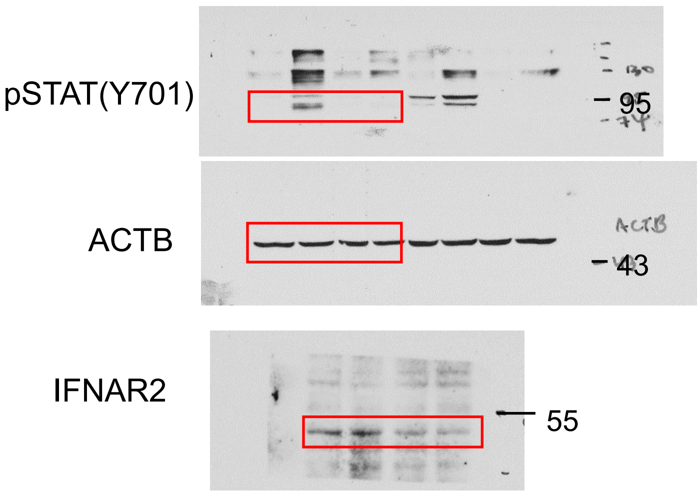

Supple Fig. 2c

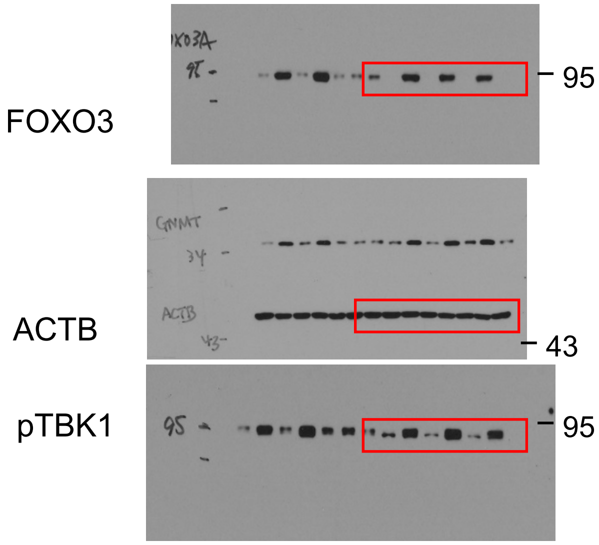

Supple Fig. 3a, left

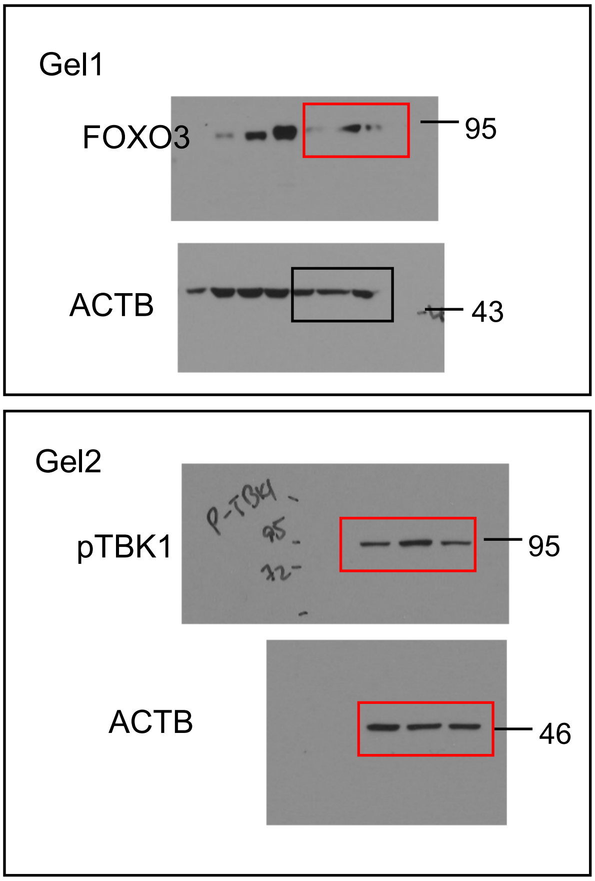

Supple Fig. 3a, right

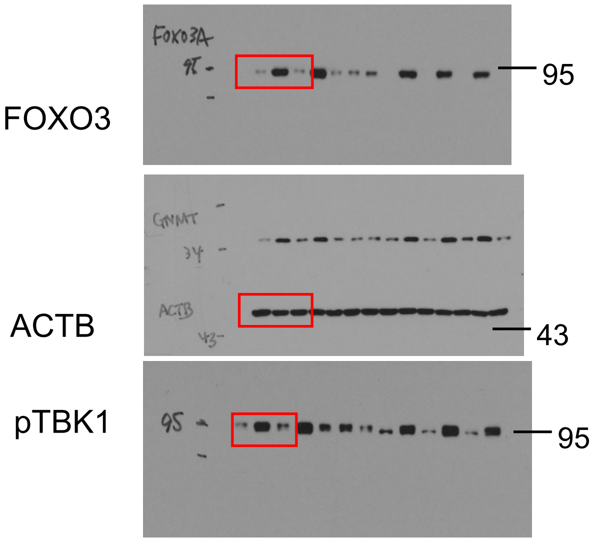

Supple Fig. 3b

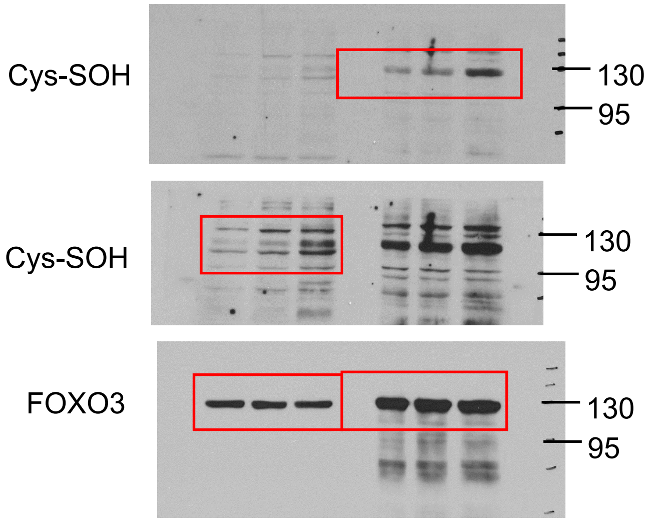

Supple Fig. 8e

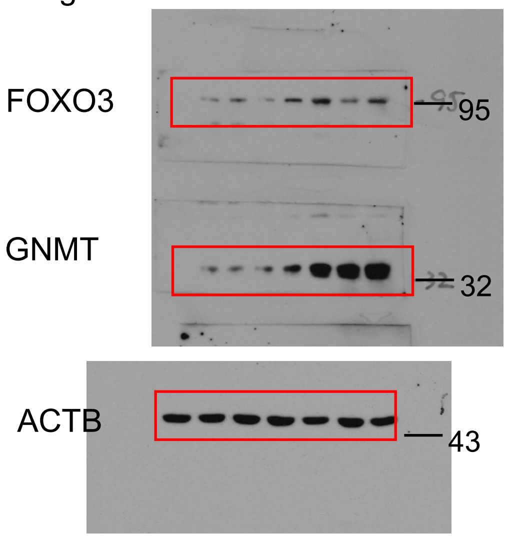

Supple Fig. 3c

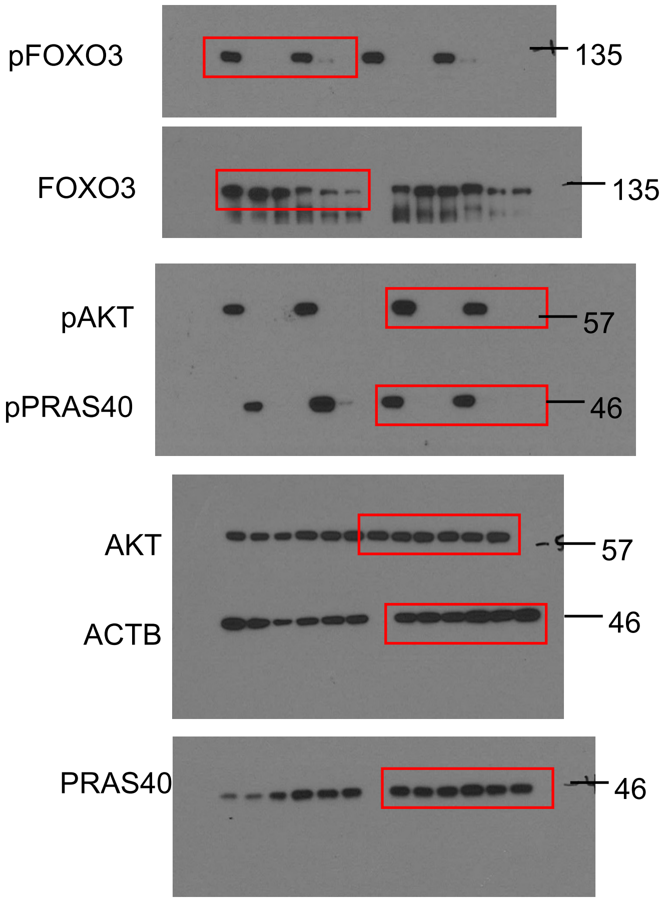

Supplement: Supplementary file 7 — Source Data [file 41467_2020_20839_MOESM7_ESM.zip › Source data/Westernblot uncroped gels.pdf]
